# INNOVATION IN AGING & ACQUIRED BRAIN INJURY REHABILITATION: THE POTENTIAL OF MIXED REALITY TECHNOLOGIES

**DOI:** 10.1093/geroni/igad104.3435

**Published:** 2023-12-21

**Authors:** Mathieu Figeys, Farnaz Koubasi, Doyeon Hwang, Allison Hunder, Andrew Chan, Antonio Miguel-Cruz, Adriana Ríos Rincón

**Affiliations:** University of Alberta, Edmonton, Alberta, Canada; University of Alberta, Edmonton, Alberta, Canada; University of Alberta, Edmonton, Alberta, Canada; University of Alberta, Edmonton, Alberta, Canada; Glenrose Rehabilitation Hospital, Alberta Health Services, Edmonton, Alberta, Canada; University of Alberta, Edmonton, Alberta, Canada; University of Alberta, Edmonton, Alberta, Canada

## Abstract

**Background:**

Every 21 seconds an American experiences a Traumatic Brain Injury (TBI), and every 40 seconds, another endures a stroke. These events fall under Acquired Brain Injuries (ABI) with prevalence rates impacted by an aging population. ABI can lead to various physical, cognitive, and mental impairments, emphasizing the need for innovative rehabilitation strategies, including those specific to older adults. Mixed Reality (MR) technologies offer potential in enhancing ABI rehabilitation, yet face challenges such as methodological inconsistencies, differing clinical populations, and exaggerated efficacy claims.

**Objectives:**

1) Review MR’s role in ABI rehabilitation among older adults, assessing the implications of aging, clinical, and technological considerations. 2) Present ongoing MR research at the Glenrose Rehabilitation Hospital (GRH, Edmonton, Canada).

**Methods:**

1) A systematic review following PRISMA guidelines was performed across seven databases, with two independent reviewers analyzing the data. The analysis emphasized clinical objectives, MR systems, levels of evidence, and technology readiness levels. 2) QR codes that link to videos will highlight the ongoing research and development of MR-delivered ABI rehabilitation at the GRH.

**Results:**

Twenty-six studies met the inclusion criteria, totalling 453 subjects with ABI (mean age: 60 ± 5.34 years). MR applications mainly targeted upper limb motor rehabilitation, revealing an overall low level of evidence and a median technology readiness level of 6 (prototypes tested in relevant environments).

**Conclusion:**

Despite existing variability and technological challenges, the promising results stress the importance of ongoing research and innovation in MR rehabilitation. The GRH stands as a key research hub, actively advancing this field.

